# Detection of intravenous leiomyomatosis with intracardiac extension by ultrasonography: A case report

**DOI:** 10.3892/ol.2013.1387

**Published:** 2013-06-10

**Authors:** WEI LIU, MING LIU, JING XUE

**Affiliations:** 1 Department of Gynecology, Shandong Provincial Hospital Affiliated to Shandong University, P.R. China; 2 Department of Gynecology, Shandong University, Jinan, Shandong 250012, P.R. China

**Keywords:** intravascular growth, intracardiac leiomyomatosis, ultrasonography, diagnostic imaging

## Abstract

Intravenous leiomyomatosis (IVL) is characterized by histologically benign tumors that exhibit aggressive clinical behavior. On rare occasions, the tumors may extend into the regional and systemic veins, thus reaching the heart. This may subsequently cause intracardiac leiomyomatosis (ICL), which may lead to congestive heart failure and occasionally, sudden fatalities. Due to its rarity and diffuse symptoms, the misdiagnosis of ICL is common and as a result, the condition may be under-reported. The present study reports a 33-year-old female who was admitted to Shandong Provincial Hospital Affiliated to Shandong University for myomectomy due to a rapidly growing myoma of the uterus. In routine pre-operative abdominal ultrasonography, a moderately sized echoic mass in the right internal iliac vein was observed, which extended to the common iliac vein, the inferior vena cava and the orifice of the right atrium. A presumptive diagnosis of ICL was made. The patient underwent a well-prepared one-stage thoraco-abdominal surgical procedure and the pathological report confirmed ICL. This case illustrates that the early detection of ICL may prevent a potential emergency situation and abdominal ultrasonography may be considered a useful tool in the diagnosis of ICL.

## Introduction

Intravenous leiomyomatosis (IVL) is a rare clinical entity characterized by the intraluminal extension of leiomyoma pedicles into the regional and systemic veins. Typically, the tumor enters the uterine veins and may progressively extend to the iliac vein, the inferior vena cava and occasionally to the right atrium (1). Lesions may extend into the heart, generating a condition known as intracardiac leiomyomatosis (ICL), which may lead to congestive heart failure and occasionally sudden fatalities. To date, >100 cases of ICL have been reported in the literature. In five of these cases, mortality occurred due to right heart obstruction (2). Due to its rarity and subtle clinical features, the misdiagnosis of ICL is common in clinical practice and may lead to delayed treatment and fatal outcomes. The present study reports a case of ICL that presented with no specific symptoms and was diagnosed by routine abdominal ultrasonography (US) prior to surgery for uterine leiomyoma. Thus, pre-operative assessments using abdominal US in these cases may be considered extremely important tools of diagnosis (3). Written informed consent was obtained from the patient.

## Case report

A 33-year-old, gravida 2 para 1 patient was admitted to Shandong Provincial Hospital Affiliated to Shandong University (Shandong, China) for myomectomy due to a rapidly growing myoma of the uterus. The patient’s medical history included hypertension and Hashimoto’s thyroiditis. The patient reported pelvic pain one month prior to the hospital admission and a clinical examination revealed a mobile, enlarged uterus with myoma. The patient reported no weight loss or cardiopulmonary symptoms. Four years earlier, and in association with a pregnancy, the myoma had measured 5 mm in size. The myoma was approximately the same size when observed during an abortion two years later. Vaginal US revealed a mass of 86×74×45 mm in size on the right side of the uterus. The results of a number of laboratory examinations, including those for tumor marker levels, were normal. Electrocardiography (ECG) demonstrated a sinus pattern with ST depression. X-rays of the thorax indicated a normal cardiac silhouette and a normal appearance to the lungs.

**Figure 1.**
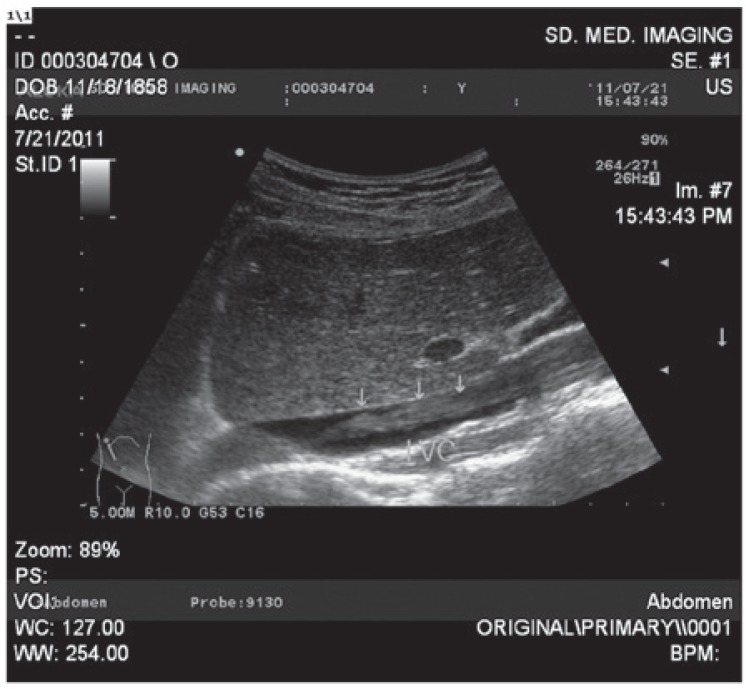
Abdominal US revealing a mobile solid mass in the inferior vena cava (IVC), which extended to the orifice of the right atrium. US, ultrasonography.

Routine pre-operative abdominal US revealed a moderately sized echoic mass in the right internal iliac vein. The mass extended to the common iliac vein, the inferior vena cava and the orifice of the right atrium (Fig. 1). The common iliac vein was dilated to 20 mm in diameter on the right side compared with 12 mm on the left side. The mass had well-demarcated borders and was not attached to the vessel walls. Magnetic resonance imaging (MRI) demonstrated a large mixed-signal intramural mass on the right side of the uterus and a mass with a similar signal extending to the right atrium via the right iliac vein and inferior vena cava. The initial diagnosis was of ICL. Following careful preparation, the patient underwent a one-stage thoraco-abdominal surgical procedure with a total hysterectomy, bilateral salpingo-oophorectomy and removal of the ICL pedicle, which measured 630 mm in length and 5 mm in diameter, together with several smaller pedicles. The pathology report confirmed the diagnosis of ICL. The patient was discharged following an uneventful post-operative course. No signs of recurrence occurred in the following 13 months.

## Discussion

IVL with intracardiac extension is an extremely rare type of benign tumor associated with a high rate of mortality (2). The etiology of the disease is not yet fully understood. In total, ∼90% of reported cases have occurred in parous females and 10% of patients have presented with a history of previous pelvic surgery and hysterectomy (4). It has been suggested that an incomplete hysterectomy may promote the proliferation of IVL (5), or alternatively, that IVL may originate in the smooth muscle cells of the vessel wall (6). The present study patient had a normal pregnancy and delivered via cesarean section four years earlier. This was not followed by growth of the myoma. However, growth was initially observed following an abortion two years earlier, which may have been stimulated by either the pregnancy or the abortion. Hormones have been suggested to stimulate IVL (7).

The clinical course of IVL is variable and dependent on the burden of the disease. It is most commonly observed in middle-aged females (5) and in conjunction with leiomyoma of the uterus or an ovarian tumor (8). Two-thirds of patients exhibit diffuse symptoms, including pelvic discomfort or abdominal pain; however, IVL may also be diagnosed accidentally during abdominal US, unrelated surgeries or at autopsy (9). As IVL extends to the larger veins and the right atrium (ICL), symptoms of impaired venous circulation develop, including Budd-Chiari syndrome (10), congestive heart failure and sudden fatalities (8). Although the lesion reached the orifice of the right atrium in the current study, the patient presented with no symptoms of impaired circulation. This may have been as the diameter of the tumor (5 mm) was not large enough to significantly affect the blood circulation.

A diagnosis of ICL is usually made at the time of surgery. Abdominal US, computed tomography, MRI and echocardiography possess different advantages in identifying IVL and ICL. In the present case, IVL was suspected following abdominal US and confirmed by MRI. Detailed pre-operative information with regard to the localization and extension of the tumor is essential for a successful outcome (11).

Surgical treatment with extensive resection has been demonstrated to provide the optimal mid- and long-term prognosis (9). The procedure should include removal of the intravenous tumor extension and total hysterectomy and bilateral salpingo-oophorectomy, as the tumor is considered to be estrogen-dependent (12). Anti-estrogenic drugs have been used pre- and post-operatively to reduce the tumor burden and control residual tumors (13).

Histologically, IVL resembles a typical leiomyoma and the rate of mitosis is low. Perinodular hydropic degeneration in a myoma (which was identified in our patient) may be a precursor of IVL (14), thus physicians should be aware of this when diagnosing IVL.

For middle-aged females with rapidly growing myomas and a history of pelvic surgery, IVL and ICL should be considered. Abdominal US is essential for the pre-operative assessment of ICL.
